# Classification of proximal tibial fractures in children

**DOI:** 10.1007/s11832-009-0167-8

**Published:** 2009-03-17

**Authors:** Scott J. Mubarak, Jung Ryul Kim, Eric W. Edmonds, Maya E. Pring, Tracey P. Bastrom

**Affiliations:** 1 Pediatric Orthopedics and Scoliosis Division, Rady Children’s Hospital and Health Center, 3030 Children’s Way, Suite 410, San Diego, CA 92123 USA; 2 Department of Orthopedic Surgery, College of Medicine, Research Institute of Clinical Medicine, Chonbuk National University Hospital, Jeonju, South Korea

**Keywords:** Proximal tibia, Fracture, Classification

## Abstract

**Purpose:**

To develop a classification system for all proximal tibial fractures in children that accounts for force of injury and fracture patterns.

**Methods:**

At our institution, 135 pediatric proximal tibia fractures were treated from 1997 to 2005. Fractures were classified into four groups according to the direction of force of injury: valgus, varus, extension, and flexion–avulsion. Each group was subdivided into metaphyseal and physeal type by fracture location and Salter–Harris classification. Also included were tibial tuberosity and tibial spine fractures.

**Results:**

Of the 135 fractures, 30 (22.2%) were classified as flexion group, 60 (44.4%) extension group, 28 (20.8%) valgus group, and 17 (12.6%) varus group. The most common type was extension-epiphyseal-intra-articular-tibial spine in 52 fractures (38.5%). This study shows that proximal tibial fractures are age-dependent in relation to: mechanism, location, and Salter–Harris type. In prepubescent children (ages 4–9 years), varus and valgus forces were the predominate mechanism of fracture creation. During the years nearing adolescence (around ages 10–12 years), a fracture mechanism involving extension forces predominated. With pubescence (after age 13 years), the flexion–avulsion pattern is most commonly seen. Furthermore, metaphyseal fractures predominated in the youngest population (ages 3–6 years), with tibial spine fractures occurring at age 10, Salter–Harris type I and II fractures at age 12, and Salter–Harris type III and IV physeal injuries occurring around age 14 years.

**Conclusion:**

We propose a new classification scheme that reflects both the direction of force and fracture pattern that appears to be age-dependent. A better understanding of injury patterns based on the age of the child, in conjunction with appropriate pre-operative imaging studies, such as computer-aided tomography, will facilitate the operative treatment of these often complex fractures.

## Introduction

Fractures around the proximal tibial growth plate are relatively infrequent injuries, with most resulting in anterior, anterolateral, and anteromedial epiphysis displacement relative to the metaphysis [1]. The tibial tuberosity acts as an effective block, making posterior displacement rare. In such injuries, the fracture usually propagates along the physeal extension beneath the tibial tuberosity, so that the proximal tibial epiphysis and tuberosity are displaced as a single unit [2]. Fractures of the proximal tibial metaphysis usually occur in children of ages 3–6 years, and may be complete or greenstick. Most often, the medial cortex of the fracture is gapped open, producing a valgus deformity [3–6].

Fractures of the pediatric proximal tibia can be caused by a direct or indirect force. A direct force may be imposed when a child’s leg is run over by the wheel of a vehicle, when it is caught between the bumpers of two automobiles, or struck by a bicycle pedal. However, an indirect mechanism is more common. The lower leg is forced into abduction, adduction, or hyperextension against a knee fixed in space. Indirect injuries to adolescents occur during sporting activities, motor vehicle accidents, or even falls. Less frequently, tibial tubercle injuries have been described in boys, nearly all aged 15 or 16 years, as they initiated or landed from a jump [7]. Because of this finding, some authors have reported that avulsion fractures of the tibial tubercle should constitute a distinct group of fractures, considered separate from standard fracture patterns [2, 8]. Tibial spine avulsion occurs when an axial loaded knee undergoes hyperextension and the femur externally rotates [9, 10].

There is no comprehensive classification system for pediatric proximal tibial fractures [26–28]. Current classification systems are limited to one particular type of fracture (i.e., tibial tuberosity, tibial spine, or Salter–Harris physeal injury) and do not account for all fracture patterns in both anteroposterior and lateral planes. At the distal end of the tibia, there are multiple comprehensive systems of fracture classification, including the Lange–Hansen classification. These types of comprehensive classifications provide two major benefits to the treating orthopedic surgeon. One benefit is that these classifications highlight the mechanism of injury as it relates to the fracture pattern, and this facilitates patient care by providing an understanding towards the reduction of those fractures. The second major benefit is that these classifications provide knowledge on the likely fracture patterns based on mechanism and other factors, which will limit the number of missed injuries and decrease the complications associated with those missed injuries. We propose a classification scheme for the proximal tibia that reflects both direction of force and fracture pattern that facilitates patient care and decision-making by highlighting those patterns as they relate to patient age.

## Materials and methods

Following Institutional Review Board approval, a retrospective review of 135 patients treated at our institution for pediatric proximal tibial fractures between January 1997 and February 2005 was completed. All medical records, including emergency room evaluation and treatment, radiographs, operation reports, anesthesia records, and clinic notes, were reviewed. All patients were treated by fellowship-trained pediatric orthopedists. Data collection included: gender, age, extremity, and cause of injuries. Radiographic evaluation included: mechanism of injury, fracture displacement on either the anteroposterior and lateral radiograph, and evidence of angular deformity.

**Fig. 1:**
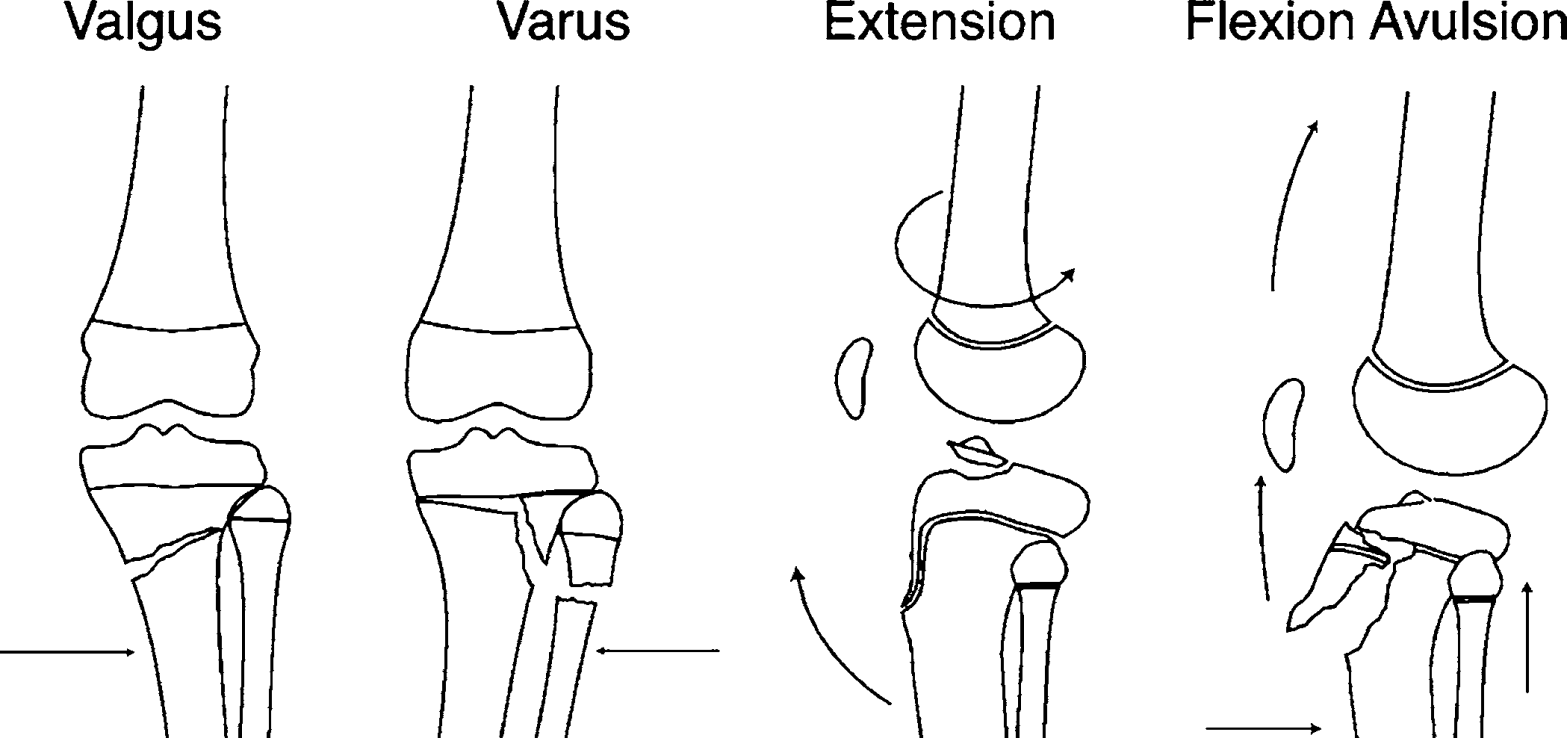
Classification scheme for pediatric proximal tibial fractures

Fractures were classified into four groups according to the direction of force of injury; flexion–avulsion, extension, valgus, varus (Fig. 1). The direction of force was determined based on the initial radiographs and the location of the intact and open cortices. Each group was subdivided into metaphyseal and physeal fractures. The physeal injuries were further divided into extra-articular (Salter–Harris type I and II) and intra-articular (Salter–Harris type III, IV, and tibial spine) fractures. We further stratified the mechanism of injury based on the age of the children. Differences in age for the direction of force of injury and for the location of fracture were compared using analysis of variance (ANOVA, *P* < 0.05, SPSS Inc., Chicago, IL, USA). Data were checked for normality and homogeneity of variances. Bonferroni post hoc tests were performed for multiple comparisons.

## Results

**Fig. 2:**
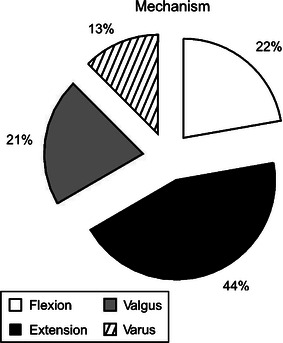
The frequency of pediatric proximal tibial fractures classified using our system

The initial injury radiographs of 135 patients were reviewed. The fractures were classified using our comprehensive classification system (Fig. 2). Of the 135 patients with injury data, 70 fractures were left-sided and 65 were right-sided. There were 96 boys and 39 girls. In most categories, boys accounted for a much greater percentage, except for the valgus and varus metaphyseal type in the younger children, when girls predominated.

**Fig. 3:**
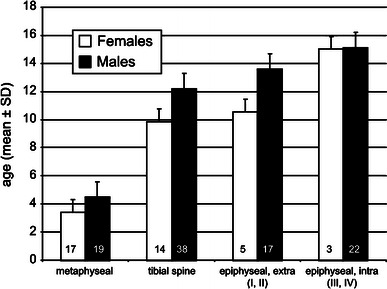
Age and gender distribution for extension injuries demonstrating an age-dependent relationship to fracture location and Salter–Harris type

The mechanism of injury, location of injury, and Salter–Harris type were all found to be age-dependent for proximal tibial fractures. All three groups (extension, flexion, varus/valgus) differed significantly from one another (*P* ≤ 0.001). In children (ages 4–9 years), the predominate mechanism of injury was either a varus or valgus force. Around age 10–12 years, an extension force that usually occurred during a sporting activity was the most common. After age 13 years, the flexion–avulsion mechanism/pattern is seen primarily, particularly in boys. There is a clear delineation in the location of fractures observed in the various age groups as well (Fig. 3). Metaphyseal fractures tended to occur around age 3–6 years (mean age 3.8 years), tibial spine fractures at age 10, Salter–Harris type I–II fractures at age 12, and Salter–Harris type III–IV physeal injuries around age 14 years. The metaphyseal group was significantly younger than the tibial spine and two Salter–Harris type groups (all *P* ≤ 0.001). The tibial spine group did not differ significantly from the Salter–Harris type I–II fractures (*P* = 0.056), but was significantly younger than the Salter–Harris type III–IV fractures (*P* ≤ 0.001). The Salter–Harris type I–II group was also significantly younger than the Salter–Harris type III–IV group (*P* = 0.007).

The most common cause of injuries, by frequency, in our population was: sports-related in 59 fractures (43.7%), fall from a height in 28 fractures (20.7%), fall from a bicycle or motorcycle in 28 fractures (20.7%), and motor vehicle accidents in 19 fractures (14.1%).

**Fig. 4:**
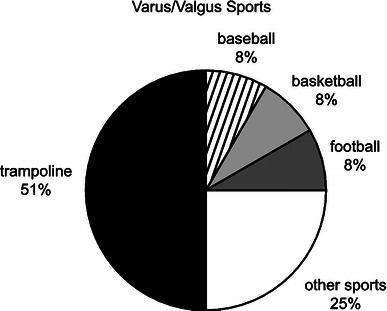
Distribution of varus/valgus sports-related injuries

**Fig. 5:**
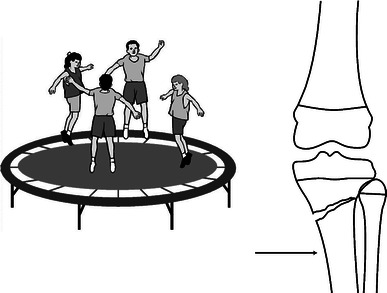
Trampoline jumping (often with multiple children on the trampoline) was the most common activity producing the greenstick valgus metaphyseal fracture

In the entire series of the fractures, 28 (21%) were produced by a valgus mechanism and 17 (13%) by varus mechanism. If we combine varus and valgus fractures, they account for 34% of the proximal tibia fractures. Most were metaphyseal and the average age of injury was 7 years. In this combined group, trampoline injuries accounted for half of the ‘sports’-related injuries, and competitive sports such as basketball, football, and baseball accounted for one-fourth of the varus/valgus injuries (Fig. 4). The most common fracture pattern was the valgus greenstick (Cozen’s) metaphyseal fracture. The prototypic activity was trampoline jumping (Fig. 5). As the age of the child advanced, the location of the fractures shifted to become more proximal, to the point of involving the physis at an average age of 12 years.

**Fig. 6:**
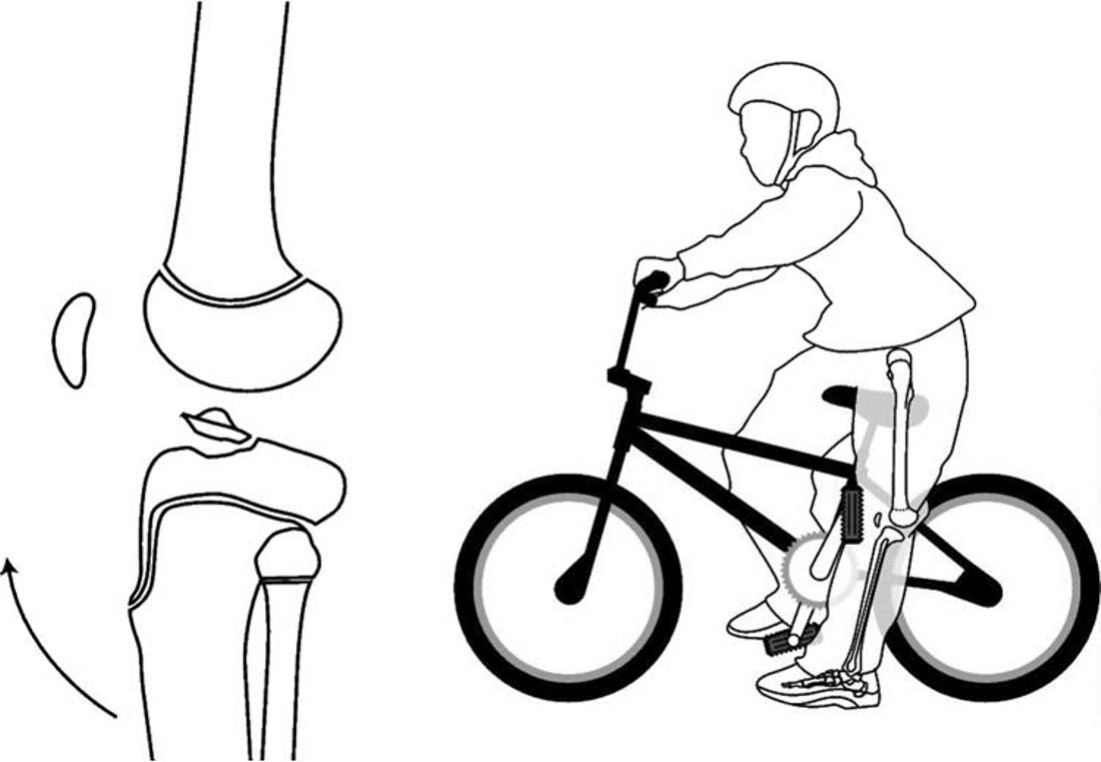
Hyperextension injuries were commonly seen with the use of a bicycle, particularly when attempting to brake urgently with the ipsilateral foot striking the ground

The third mechanism of fracture was hyperextension, and this proved to be the largest group of fractures (44%). The average overall age in this group was 11 years. In children less than 6 years old, the fractures went through to the metaphysis. At around ages 10 years for girls and 12 years for boys, the tibial spine injuries (52 fractures) occurred with the prototypic activity being the use of their foot on the ground to brake while riding a bicycle (Fig. 6). Similarly, the advancing age of the child in this mechanism group affected not only the fracture location, but also the location of the fracture in relation to the growth plate. Salter–Harris type I and II fractures occurred at a mean age of 13 years, and for those a year older (mean age of 14 years), type III and IV fractures are most common. Importantly, neurovascular injuries may occur in this latter group (Fig. 4).

**Fig. 7:**
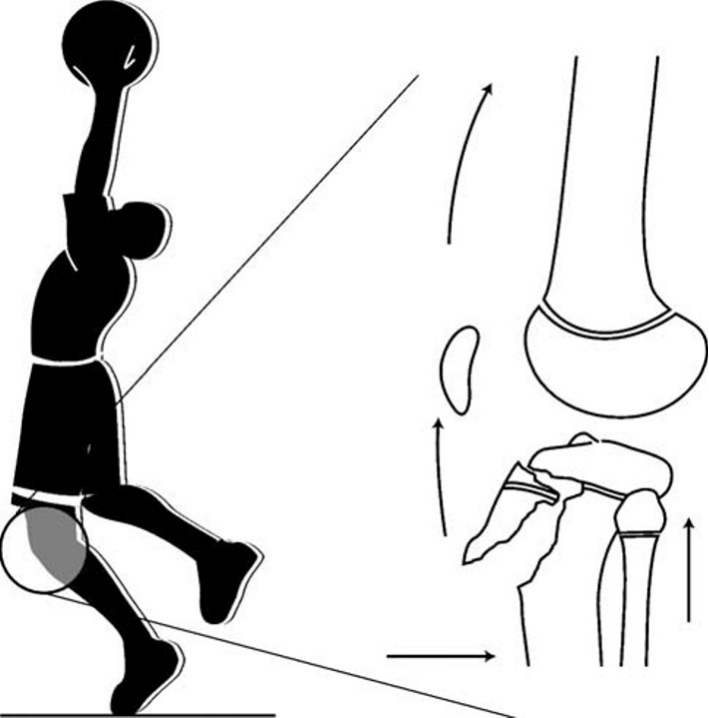
Flexion–avulsion injuries are most common in adolescent boys, particularly when attempting to push off for a jump in a sport such as basketball

The fourth mechanism is flexion–avulsion, and this type occurred primarily during adolescence (mean age of 15 years) and accounted for 22% of the fractures. The prototypic sport was basketball and involved only boys 100% (10). In interviewing our patients, most described injuring themselves while attempting a jump, such as during a basketball lay-up or dunk. Their stable base leg was flexed for a power push-off as they approached the net and, as they initiated the push-off (forced quadriceps contraction with knee flexed), they all felt a pop at that moment; the avulsion fracture occurred at this point in time and the jump never actually takes place (Fig. 7).

Compartment syndromes were encountered in three patients: one child with a flexion–avulsion type (tibial tuberosity injury), one with a valgus type, and the other with an extension type (Salter–Harris type II) that was associated with a concomitant severe artery and nerve injury.

## Discussion

The incidence of proximal tibial physeal fractures is from 0.6 to 2.1% of all physeal injuries [11]. This low incidence can be attributed to the intrinsic anatomical stability of the proximal tibial epiphyseal/physeal region. The lateral side is buttressed by the proximal fibula, and on the medial side, the superficial portion of the medial collateral ligament inserts not on the epiphysis but on the proximal metaphysis distal to the physis. The majority of these injuries result from an indirect force, such as jumping and landing awkwardly.

This study shows that mechanism, location, and Salter–Harris type of proximal tibial fractures are age-dependent (Fig. 3). We believe that the location of injury and growth plate involvement are related to the child’s age because, with maturation of the musculo-skeletal system, the weak link on the proximal tibia shifts from a more metaphyseal location towards the tibial eminence and then to the physis with skeletal maturity. Moreover, the activities in which the children engage require greater force generation of muscles on bone for their sports participation. This change is related to both the augmented level of play and also to the increased body mass of the child, thereby, shifting the mechanism of injury in an age-dependent fashion.

Varus and valgus mechanisms of injury predominate the youngest group (age 4–9 years), whereas a mechanism of forced extension, particularly with sporting activities, predominates the 10–12 years old age group. Finally, children greater than 13 years old, especially boys, are prone to succumb to a flexion–avulsion mechanism of injury. Along those lines, the youngest group of children tend to sustain metaphyseal fractures and 10-year-old children get tibial spine fractures, whereas children near age 12 years of age are prone to have Salter–Harris type I and II (extra-articular) fractures and the oldest group of children (around 14 years of age) develop Salter–Harris type III and IV (intra-articular) physeal injuries.

### Varus and valgus fractures

Fractures of the proximal tibial metaphysis occurred in very young age (mean age 3.8 years) and the mechanism of injury is similar to that of proximal tibial epiphyseal fractures. The most common mechanism of injury was valgus, followed by varus, extension, and flexion. Most of these fractures occurred through the metaphysis at the level of the tibial tubercle. The age range of the children is 3–6 years. The prototypic activity in this series was trampoline jumping (Fig. 5). The mechanism of injury is forced valgus, varus, or, occasionally, hyperextension against the fixed knee. Usually, the force is applied to the lateral aspect of the extended knee, which causes the cortex of the medial metaphysis to fail in tension. This produces the greenstick valgus metaphyseal fracture.

In our review of these 135 fractures, we did not encounter any varus, Salter–Harris type I or II fractures. We hypothesize that the reason for this is that the proximal fibula acts as a buttress on the lateral side of the tibia and that the tibial tuberosity effectively blocks any potential posterior displacement. The importance in understanding this fracture pattern is that the long-term outcome risks a genu valgum deformity [4–6, 12–14]. Therefore, observation and potential surgical intervention may be warranted in the treatment of these children.

### Extension fractures

We grouped tibial spine fractures in the extension mechanism category, since tibial spine avulsions occur when an axial loaded knee undergoes hyperextension and the femur externally rotates. Also, a blow to the front of the fixed knee may drive the femur posteriorly on the fixed tibia and result in this avulsion fracture of the tibial spine. The most common indirect force seen in this study, and in the literature [9], was the use of a bicycle (especially when attempting to brake urgently with the ipsilateral foot striking the ground) (Fig. 6).

The injuries to the tibial eminence, or spine, have been classified by Meyers and McKeever [9] and was further expanded to include four distinct types of fracture: nondisplaced, partial displacement, complete displacement, and complete displacement with comminution [15]. These fractures can be treated as closed if reduction is successful, but should otherwise undergo operative reduction due to the potential risk to the meniscus [16] and the risk towards long-term laxity secondary to attenuation of the anterior cruciate ligament prior to fracturing [17, 18].

Not all fractures sustained via an extension mechanism resulted in a tibial eminence fracture; yet, the other type of fractures sustained by an extension mechanism, usually a direct force, continued to be age-dependent. In children less than 6 years old, the fracture was usually through the metaphysis, whereas this same mechanism in the early teens would result in a fracture through the physis. The popliteal artery and nerve are at risk with this mechanism in any of the age groups evaluated.

### Flexion–avulsion fractures

A direct flexion force at the proximal tibia is unlikely to be deforming, since the knee is able to absorb that energy with genu-flexion. However, indirect injuries of flexion with resultant avulsion of tissue seem to occur especially in adolescence and during sporting activities or falls. The flexion–avulsion injuries described here tend to be sustained by boys aged 14–16 years. Watson-Jones [8] initially described three types of avulsion fractures of the tibial tubercle. Ogden et al. [2] refined that initial work and described three types of fractures that differ in the distance of the fracture from the distal tip of the tubercle. Neither of these classification systems accounts for all flexion type fractures seen at the proximal tibia. Kanellopoulos et al. [19] reported a type of triplane fracture of the proximal tibia. Donahue et al. [20] reported on two cases of combined physeal and apophyseal fractures of the proximal tibia with anterior angulation from an indirect force. These injuries represent a combination of a Salter–Harris type II or III fracture of the proximal tibial physis and a tibial tubercle avulsion. They modified the Watson-Jones/Ogden classification by adding a type IV variant that encompasses this combined fracture pattern [2, 21–23].

We believe this indirect, flexion–avulsion type fracture occurs as the teenage boy initiates a jump. The quadriceps mechanism exerts a strong eccentric muscular contraction on a flexed knee as the athlete begins his jump. The subsequent force avulses the tibial tubercle with or without extension into the joint, stopping the jump from ever taking place (Fig. 7). This mechanism may result in a simple apophyseal fracture of the tibial tubercle or, depending on skeletal maturity, they may combine with a physeal injury of the proximal tibia, resulting in a type of triplane fracture.

### Associated injuries

Acute complications of proximal tibial fractures may include arterial injuries, nerve injuries, and compartment syndrome. A compartment syndrome associated with tibial tubercle avulsion was first reported by Polakoff et al. [24] in 1986. The anatomy of the proximal tibia and the tibial tubercle with nearby branches of the anterior tibial recurrent artery is a predisposing factor for the development of compartment syndrome [25]. In our series, one of the eight flexion–avulsion intra-articular tibial tuberosity type fractures presented with acute compartment syndrome. We encountered two other cases of compartment syndrome; one case of a Salter–Harris type II fracture after a valgus mechanism and one case of a Salter–Harris type I fracture after an extension mechanism. All of these compartment syndromes were treated with urgent fasciotomy.

The most serious complication of these fractures is arterial disruption. The popliteal artery is supported by its major branches near the posterior surface of the proximal tibial epiphysis. Hyperextension injury that results in posterior displacement of the upper end of the tibial metaphysis may stretch and tear the tethered popliteal artery. In our series, one of the four extension mechanism (Salter–Harris type I and II) fractures had an associated arterial injury. The other three cases were mildly displaced physeal fractures. If arterial injury is suspected, then fracture fixation should be performed in conjunction with vascular surgeon consultation for potential arterial exploration and repair or vein graft. Understanding the mechanism of injury will help with understanding the associated injuries and provide help in the reduction of the fracture.

### Treatment

We propose a guideline for the treatment of proximal tibial fractures in the skeletally immature. Nondisplaced metaphyseal fractures are treated with long leg cast for 4–6 weeks. Displaced metaphyseal fractures are treated with closed reduction or open reduction under general anesthesia using the mechanism of injury. Greenstick valgus fractures are treated with varus reduction.

Nondisplaced epiphyseal fractures are treated with long leg cast for 4–6 weeks. In displaced epiphyseal fractures (including tibial spine), computed tomography is helpful and should be utilized to plan for open reduction and internal fixation performed with arthroscopic assistance or mini-open arthrotomy. Extra-articular fractures involving the physis are treated with closed or open reduction and, usually, internal fixation with a long leg cast. Displaced intra-articular and physeal fractures are treated with arthroscopic assisted open reduction and internal fixation. Understanding the mechanism of injury helps in the reduction of these fractures. Careful monitoring should be performed during the peri-treatment period for compartment syndrome and vascular injuries in any proximal tibia fractures. Long-term monitoring should also be performed via radiographic evaluation for premature physeal closure, since most of these fractures involve the growth plate of the proximal tibia.

## Conclusions

There are no comprehensive classification systems for pediatric proximal tibial fractures. Current classifications are limited to one particular type of fracture (i.e., tibial tuberosity, tibial spine) and do not account for all fracture patterns in both the anteroposterior and lateral planes. We propose a comprehensive classification scheme that reflects both the direction of force and fracture pattern to aide in treatment and associated injury awareness.

This study demonstrates that proximal tibial fractures are age-dependent in the skeletally immature when evaluating the mechanism of injury, location within the proximal tibia, and Salter–Harris type. In prepubescent children (age 4–9 years), varus and valgus forces sustained most commonly on a trampoline resulted in a metaphyseal fracture. In the juvenile group (age 10–12 years), an extension force occurring most often while riding a bicycle resulted in either a tibial spine fracture (age closer to 10 years) or a Salter–Harris type I or II fracture (age closer to 12 years). In the adolescent group (after age 13 years), the flexion–avulsion mechanism seen especially in boys playing basketball resulted in Salter–Harris type III or IV fractures. A better understanding of the injury pattern resulting in pediatric proximal tibia fractures will help with our understanding of these fractures in the skeletally immature and aide in determining the best technique of reduction required for treatment.
